# Swellings over the Limbs as the Earliest Feature in a Patient with Osteogenesis Imperfecta Type V

**DOI:** 10.1155/2014/780959

**Published:** 2014-03-18

**Authors:** Ali Al Kaissi, Rudolf Ganger, Klaus Klaushofer, Franz Grill

**Affiliations:** ^1^Ludwig Boltzmann Institute of Osteology, The Hanusch Hospital of WGKK and AUVA Trauma Centre Meidling, First Medical Department, Hanusch Hospital, Heinrich Collin Street 30, 1140 Vienna, Austria; ^2^Orthopaedic Hospital of Speising, Paediatric Department, Speisinger Street 109, 1130 Vienna, Austria

## Abstract

Swellings over the upper and lower limbs were encountered in a one-year-old child. Skeletal survey showed a constellation of distinctive radiographic abnormalities of osteoporosis, hyperplastic callus and ossification of the interosseous membrane of the forearm, femora, and to lesser extent the tibiae. Neither wormian bones of the skull nor dentinogenesis imperfecta was present. Genetic tests revealed absence of mutation in COL1A1 or COL1A2 genes, respectively. The overall phenotypic features were consistent with the diagnosis of osteogenesis imperfecta type V (OI-V). The aim of this paper is to distinguish between swellings because of intrinsic bone disorders and these due to child physical abuse.

## 1. Introduction 

Osteogenesis imperfecta is a heritable disorder characterized by bone fragility, deformity of the spine, and long bones. Additional abnormalities such as short stature, blue sclera, and dentinogenesis imperfecta have been described. Skeletal manifestations are due to a generalized deficiency of development of both membranous and endochondral bone and include markedly thinned calvarium with delayed closure of fontanelle and sutures and excessive wormian bone formation. Sillence et al. [1] developed a classification of OI subtypes: OI type I with blue sclerae; perinatal lethal OI type II, also known as congenital OI; OI type III, a progressively deforming form with normal sclera; and OI type IV, with normal sclerae. Levin et al. [2] suggested that OI subtypes could be further divided into types A and B based on the absence or presence of dentinogenesis imperfecta. In the large majority of patients with OI types I–IV, the disease is caused by mutation in the two genes that encode collagen type I alpha chains. Such mutations are absent in OI types V–VII. OI type V is characterized radiologically by interosseous membrane calcification of the forearms and a radiodense band visualised at the growth plate [3]. Patients with OI type V do not have blue sclera or dentinogenesis imperfecta. The aim of this paper is to further delineate the phenotypic characterization of osteogenesis imperfecta type V.

## 2. Clinical Report 

A-one-year-old boy was referred to our department for clinical evaluation. Progressive upper and lower limbs swellings have been noted since birth and were an unpleasant dilemma for the parents and the paediatrician. The child was a product of uneventful gestation. At birth his length, weight, and ofc were around the 25th percentile. The mother was a 28-year-gravida one-abortus 0 married to a 32-year-old unrelated man. Family history was unremarkable. His subsequent course of development has been within normal limits. At the age of one year, his length, weight, and ofc were around the 50th percentile. Craniofacially, there were sparse hair, frontal bossing, faint eye brows, depressed nasal bridge, full cheeks, and thin and transparent skin. Long philtrum was associated with thin upper lip and neither dentinogenesis imperfecta nor blue sclerae were present. The neck was short, though normal neck mobility. Mild ligamentous hyperlaxity, neither scoliosis nor kyphosis was present.

His neurological examination was normal, and there were no skin stigmata suggestive of a neurogenetic disorder. The child had normal genitalia. The following biochemical parameters were within the normal range: serum and urinary oligosaccharides, mucopolysaccharides, serum lactate, pyruvate, creatine phosphokinase, alkaline phosphatase, calcium, phosphorus, and vitamin D metabolism and chromosomal study. Hormonal investigations included thyroid hormones; adrenocorticotropic hormone and growth hormone were negative as well. Genetic testing revealed absence of mutation in COL1A1 or COL1A2 genes, respectively.

**Figure 1:**
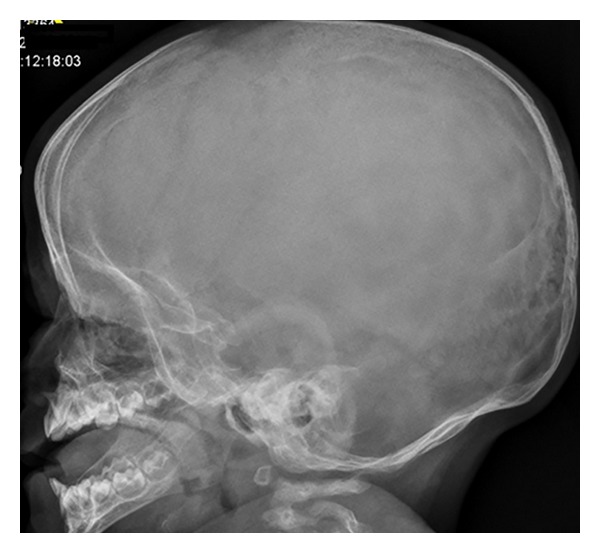
Lateral skull radiograph showed low mineralization of the skull bones associated with persistent opening of the anterior fontanelle. The brain convolutions were apparent because of the demineralized calvaria. Note the bulging frontal area. No evidence of wormian bones and/or dentinogenesis imperfecta.

**Figure 2:**
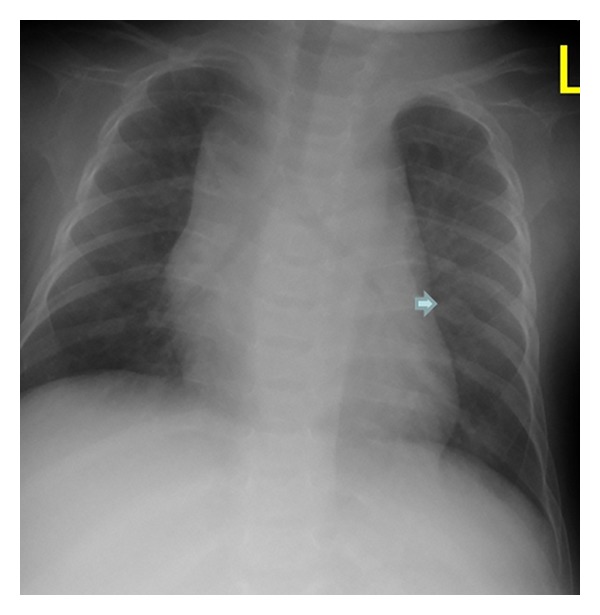
Anteroposterior thorax radiograph generalized osteoporosis associated with thin and somehow gracile and short ribs. Note callus formation on the 7th right rib (arrow).

**Figure 3:**
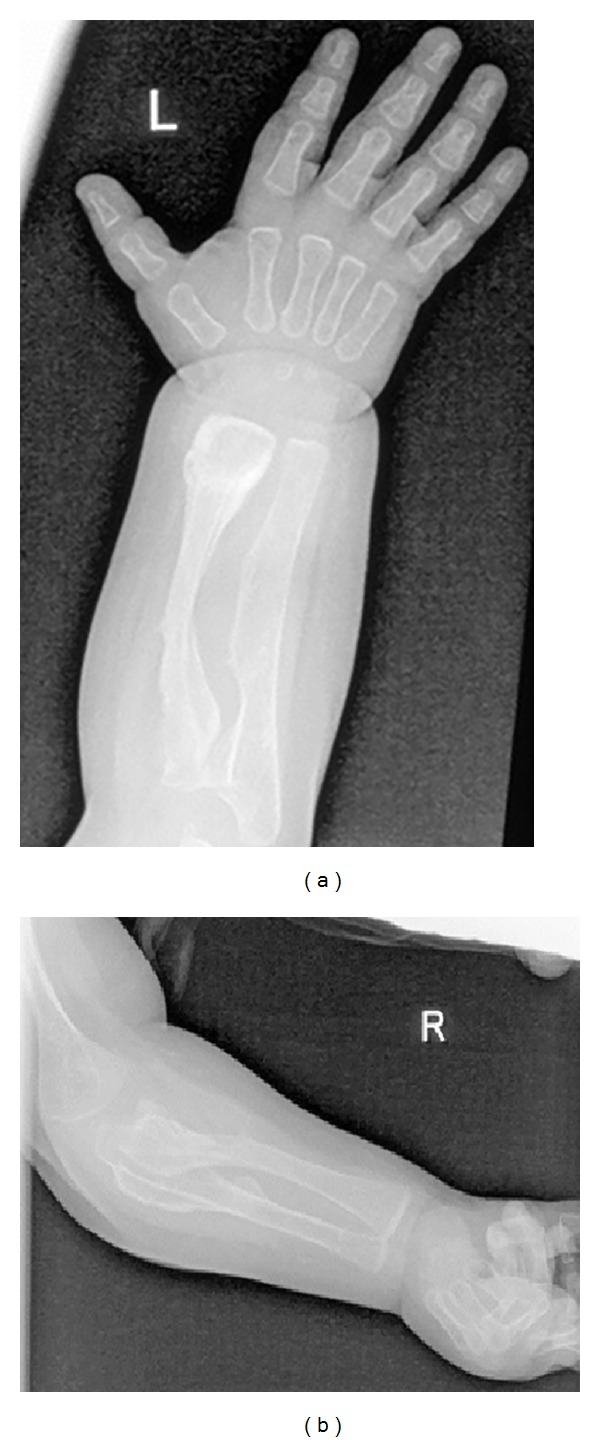
Anteroposterior upper limbs radiograph showed bilateral calcified interosseous membranes of the forearms associated with bilateral anterior subluxation of the radial heads; the ends of the long bones were thick. Osteoporosis and broad and short bones of the hands and the carpal bones were small and dysplastic associated with apparent swelling of the forearms.

**Figure 4:**
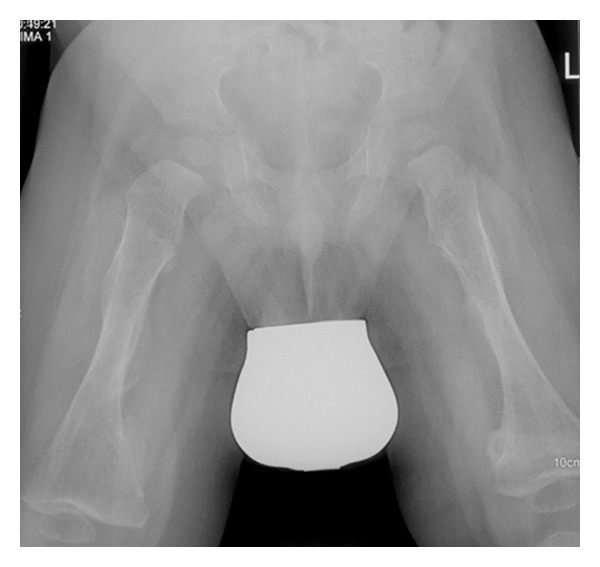
Anteroposterior pelvic and femora radiograph showed dysplastic ischium, short femora, and broad particularly over the distal parts. Calcification of the interosseous membrane was apparent bilaterally associated with overwhelming osteoporosis and the presence of a radiodense band visualized at the growth plate.

**Figure 5:**
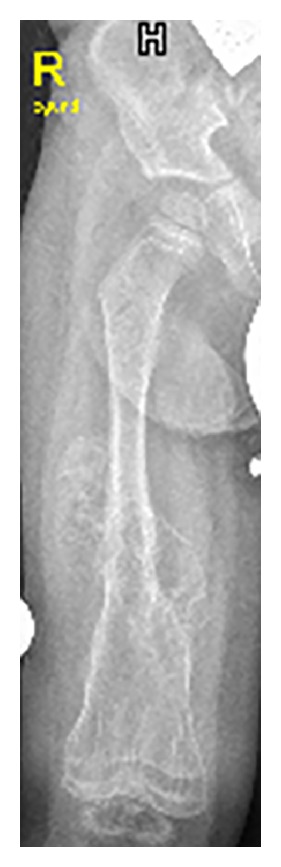
Supracondylar femoral fracture in the same patient at the age of 3 years note the profound development of callus around the lower third of the shaft of the femur. This feature looks to be a characteristic finding in patients with OI type V.

On the bases of skeletal survey showed low mineralization of the skull bones associated with persistent opening of the anterior fontanelle. The brain convolutions were apparent because of the demineralized calvaria. Note the bulging frontal area (Figure 1). The thorax showed generalized osteoporosis associated with thin and somehow gracile and short ribs (Figure 2). Bilateral calcified interosseous membranes of the forearms associated with bilateral anterior subluxation of the radial heads, and the ends of the long bones were thick. Osteoporosis and broad and short bones of the hands and the carpal bones were small and dysplastic associated with apparent swelling of the forearms (Figure 3). The pelvis showed dysplastic ischium; the femora were broad and short particularly over the distal parts. Calcification of the interosseous membrane was apparent bilaterally associated with overwhelming osteoporosis and the presence of a radiodense band visualized at the growth plate (Figure 4). Supracondylar femoral fracture in the same patient at the age of 3 years note the profound development of callus around the lower third of the shaft of the femur. This feature looks like a characteristic finding in patients with OI type V (Figure 5).

## 3. Discussion

Nonaccidental swelling over the limbs is a constant feature in children with infantile cortical hyperostosis [4]. Infantile cortical hyperostosis is a genetic disorder described by Caffey and Silverman. It is characterized by an infantile episode of massive subperiosteal new bone formation that typically involves the diaphyses of the long bones, mandible and clavicle. There is onset of pain and swelling before the age of 6 months, often in the lower limbs or jaws, and sometimes accompanied by fever. Both the clinical and radiological features usually disappear before the age of 1 year [5].

Osteogenesis imperfecta is a rare inherited disorder of connective tissue with varying degrees of severity. Orthopaedic complications include osteopenia, recurrent fractures which are prone to nonunion and malunion resulting in deformities, protrusio acetabuli, and malalignment of the limbs. Ligamentous laxity is seen frequently. In the past few decades there has been an increase in the lifespan of patients with osteogenesis imperfecta [1, 6, 7].

Osteogenesis imperfecta type I is the commonest form of osteogenesis imperfecta and is inherited as an autosomal dominant condition. Affected individuals may have blue sclerae with a tendency to fractures of the long bones, although healing occurs without deformity. In some families dentinogenesis imperfecta is a feature. The majority of patients (about 90%) with a clinical diagnosis of OI have a mutation in COL1A1 or COL1A2, the genes encoding collagen type I [8, 9]. A number of skeletal disorders can have features similar to those of OI. Osteoporosis pseudoglioma, Cole-Carpenter, and Bruck syndromes have severe bone fragility with low bone mineral content [10].

Idiopathic Juvenile Osteoporosis (IJO) is a transient nonhereditary form of bone fragility in children which is self-limiting and may be difficult to distinguish from OI type I. However collagen studies are negative in these individuals and iliac bone biopsy specimens show low remodelling rate in cancellous bone in IJO, whereas in OI there is high turnover in both cancellous and cortical bone [10].

Glorieux et al. [3] reported 7 people who might have been classified as having type V, but there was calcification of the interosseous membrane in the forearm, hyperdense metaphyses, a coarsened pattern of matrix lamellae, and a tendency to develop hyperplastic callus. Cheung et al. [11] reported a long-term followup of a child with hyperplastic callus formation and noted the considerable mobility. Type I mutations were not found. Children had moderate to severe disease often causing deformity and short stature. Teeth and sclera were normal.

Anderson Jr and Hauge [9] described the natural history of the hyperplastic callus formation in 23 patients with osteogenesis imperfecta type V. They concluded that hyperplastic callus formation is one of the conspicuous features of OI type V. The diagnosis of OI type V was based on interosseous membrane calcification of the forearms and a radiodense band visualized at the growth plate [3]. The average age of diagnosis in the above mentioned reports was 4–66 years. Exuberant callus formation in OI patients has been long described in the literature [12]. Histopathologic analyses of rapidly growing HPC have shown distinctive zones with the outer regions of callus containing oedematous region showing hypercallus trabeculae of woven bone and small cartilaginous islands [13]. Cho et al. [14] encountered a single recurrent mutation in the 5-untranslated region of IFTM5 encoding interferon-induced transmembrane protein 5.

## 4. Conclusion 

Multiple swellings over the limbs associated with radiographic features of callus-like were the reason for suspecting nonaccidental abuse. But, nevertheless, the constellation of long bone deformities and osteoporosis, with ossification of the interosseous membrane of the forearms, and anterior bilateral radial subluxation were distinctive features in favor of osteogenesis imperfecta type V.

Features of rapid periosteal apposition were noted almost all over the long bones albeit with varying degrees. The short bones of the hands were short and broad and the carpal bones were small and dysplastic. The normalcy of the parents could just as easily be a fresh dominant mutation or even an X-linked condition. Interestingly, our patient was tested for mutations affecting collagen type I, but no sequence abnormalities were detected.
